# Aberrant voxel‐based degree centrality and functional connectivity in Parkinson's disease patients with fatigue

**DOI:** 10.1111/cns.14212

**Published:** 2023-04-10

**Authors:** Aidi Shan, Heng Zhang, Mengxi Gao, Lina Wang, Xingyue Cao, Caiting Gan, Huimin Sun, Yongsheng Yuan, Kezhong Zhang

**Affiliations:** ^1^ Department of Neurology The First Affiliated Hospital of Nanjing Medical University Nanjing China

**Keywords:** degree centrality, fatigue, functional connectivity, Parkinson's disease, resting‐state fMRI

## Abstract

**Aims:**

The study aimed to investigate alterations in the inherent connectivity pattern of global functional networks in Parkinson's disease (PD) patients with fatigue.

**Methods:**

Eighteen PD patients with fatigue (PD‐F), 20 PD patients without fatigue (PD‐NF), and 23 healthy controls (HCs) were recruited and analyzed by the voxel‐wise degree centrality (DC) and the seed‐based functional connectivity (FC) analysis. Meanwhile, the surface‐based morphometry (SBM) analysis was also commanded to explore the structural alternations among groups.

**Results:**

PD‐F patients displayed reduced DC values in the left postcentral gyrus relative to PD‐NF and HCs groups, while increased DC values in the bilateral precuneus compared to HCs. Simultaneously, altered DC value in the left postcentral gyrus negatively corresponded to the mean fatigue severity scale (FSS) in PD‐F patients. Additionally, the receiver operating characteristic (ROC) curves uncovered that the reduced DC value of the left postcentral gyrus could discriminate PD‐F from PD‐NF and HCs groups. Our FC analysis further revealed that altered FC was located predominantly in the sensorimotor network in the PD‐F group. Moreover, we discovered no statistically significant differences between the three groups concerning cortical thickness.

**Conclusion:**

Our findings indicated that the altered functional connectivity in the sensorimotor network centering on the left postcentral gyrus and the bilateral precuneus might be the potential pathogenesis of PD with fatigue.

## Graphical abstract

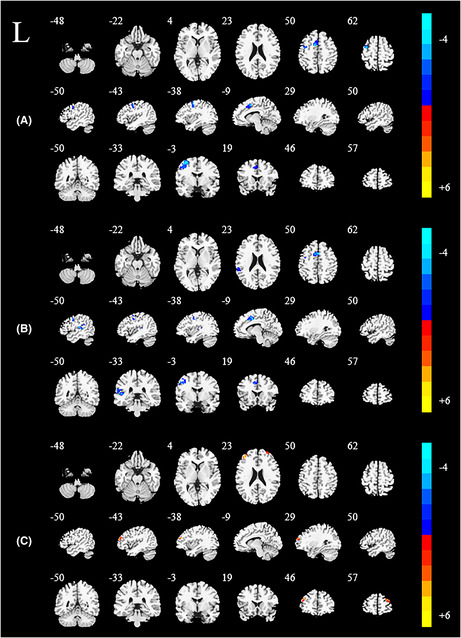

## 1 INTRODUCTION

Fatigue, a common and independent nonmotor symptom in Parkinson's disease (PD), is usually described as a feeling of exhaustion for a specific period due to unexplained drug effects, medicine, or mental disorders.^1^ According to current evidence, the prevalence of PD with fatigue (PD‐F) ranges from 33% to 58%, and the incidence of fatigue in PD patients is as high as 50%.^2^ Consequently, the high prevalence rate, as well as the damage of fatigue to patients' working hours and social activities, makes it one of the most urgent problems to solve in PD. Although fatigue is considered to be caused by the complicated interaction between the latent disease processes, peripheral control systems, central control systems, and environment‐related elements,^3^, ^4^ the exact pathophysiological mechanism of PD with fatigue remains unknown.

Concerning the pathophysiological mechanism of PD with fatigue, a growing literature has proposed that the destruction of the striato‐thalamo‐prefrontal loop may play a role in PD with fatigue.^3^, ^5^, ^6^, ^7^ Moreover, a functional imaging study showed that among patients with PD who were drug‐naïve, altered network connectivity in the sensorimotor net and default mode network (DMN) might be affiliated with fatigue.^8^ Another piece of evidence uncovered that fatigue in PD was correlated with the terrible attenuation of sensory signals from the somatosensory region to advanced motor systems.^9^ Thus, we inferred reasonably that PD patients with fatigue had atypical changes in the intrinsic connectivity pattern of brain‐wide functional networks.

Resting‐state functional magnetic resonance imaging (rs‐fMRI), offering a different perspective on neural network changes in PD, has been widely used in recently exploring the mechanism of fatigue in PD. Degree centrality (DC), a voxel‐level measurement of network connectivity, is one of the persuasive and reliable methods among nodal network metrics.^10^ DC distinguishes the node with the most connection by calculating the number of direct links to others to determine the relative importance of nodes within the network, which has been certificated anomaly in PD and related complications.^10^, ^11^, ^12^, ^13^ Therefore, we adopted the voxel‐wise DC approach to inspecting intrinsic disconnection patterns in the fatigued brains of PD patients. Furthermore, we probed the relationships between anomalous DC values of the cerebrum and fatigue severity in PD fatigue patients. We also conducted seed‐based functional connectivity (FC) analysis and surface‐based morphometry (SBM) analysis based on regions that exposed significant DC alterations to explore possible damaged networks and cortical thickness diversities of PD patients with fatigue.

## 2 MATERIALS AND METHODS

### 2.1 Subjects

Forty‐two right‐handed patients with idiopathic PD, drawn from the outpatient clinic of Neurology Department of the First Affiliated Hospital in Nanjing Medical University, were initially included in our study. Inclusion criteria were: (1) all patients obeyed the Movement Disorder Society clinical diagnostic criteria for PD^14^; (2) all patients were between the age of 40 and 80 years old; (3) all patients followed the modified Hoehn and Yahr (H&Y) rating lower than the fourth stage; (4) all patients took medicine steadily for at least 1 month. Below were the criteria for exclusion: (1) patients with unclear PD diagnosis; (2) patients with severe neurological and psychiatric diseases who could not cooperate with researchers, and those who struggled with serious respiratory, cardiovascular, and organic brain diseases; (3) patients with MRI contraindications; (4) patients with fatigue caused by taking antidepressants or other drugs; (5) patients that suffered from severe cognitive impairment (Mini‐Mental State Examination [MMSE] scores <24), apathy (Apathy scale [AS] >14) and excessive daytime sleepiness (Epworth Sleepiness Scale [ESS] >10), moderate or severe depressive and anxious symptoms (24‐item Hamilton Depression Rating Scale [HAMD‐24] >17), (Hamilton Anxiety Rating Scale [HAMA] >14); (6) Patients with excessive motion artifacts (head translation >2.5 mm or rotation >2.5°) during MRI scanning. Four patients with excessive head motion were excluded, accordingly. At the same time, 23 healthy controls (HCs) with similar age, gender, and educational levels were also enlisted. Likewise, the HCs group was also subject to exclusion criteria.

This study surrendered the Declaration of Helsinki and was approved by the ethics committee of the First Affiliated Hospital of Nanjing Medical University. In addition, all subjects signed written informed consent.

### 2.2 Clinical assessment

Parkinson's disease patients were categorized with fatigue (*n* = 18, average FSS >4) and without fatigue group (*n* = 20, average FSS ≤4) by fatigue severity scale (FSS). FSS, a 9‐item questionnaire, asks participants to mark the severity and impact of fatigue on daily life on a 1–7 scale (strongly disagree to strongly agree) and has been widely used in PD fatigue‐related research.^15^ In addition, the modified H&Y stage and Unified Parkinson's Disease Rating Scale III (UPDRS‐III) were used to evaluate the stage of disease and severity, respectively. MMSE was used to measure the overall cognitive state, while HAMD and HAMA to assess depression and anxiety, ESS to evaluate sleep disorders,^16^ and AS to estimate the apathetic state.^17^ Moreover, we computed the levodopa equivalent daily dose (LEDD) for patients involved to judge the use of dopaminergic drugs.^18^ Notably, all clinical scales and MRI data acquisition were carried out at least 12 h after the drug's withdrawal to avoid possible pharmacological interferences in our experiment.

### 2.3 MRI data acquisition

We performed MRI examination using the Siemens 3.0‐Tesla signal scanner (Siemens Medical Solutions) equipped with an eight‐channel phased array head coil. During the scan, foam padding and earplugs were prepared to restrict the motion of the head and curtail scanner noise. A further requirement was that all subjects kept still, shut their eyes, and did not think about anything special during the examination. T1‐weighted and sagittal 3D magnetization‐prepared rapid gradient echo (MP‐RAGE) sequences obtained high‐resolution brain structural images with parameters as follows: repetition time (TR) = 1900 ms, echo time (TE) = 2.95 ms, flip angle (FA) = 9°, slice thickness = 1 mm, slices = 160, field of view (FOV) = 230 × 230 mm^2^, matrix size = 256 × 256, and voxel size = 1 × 1 × 1 mm^3^. Functional images were collected with an echo‐planar imaging (EPI) sequence with the following parameters: TR = 2000 ms, TE = 21 ms, FA = 90°, FOV = 256 × 256 mm^2^, in‐plane matrix = 64 × 64, slices = 35, slice thickness = 3 mm, slice gap = 0 mm, voxel size = 3 × 3 × 3 mm^3^, total volumes = 240.

### 2.4 Preprocessing of fMRI data

Imaging data were preprocessed and analyzed on DPABI software (http://www.restfmri.net/forum/dparsf). Steps were divided into the following aspects: First, to eliminate the interference of unstable magnetic field and surrounding environment, we discarded the image data of the first 10 time points and corrected the remaining 230 images for the slice timing and head motion (Friston 24 parameter). One PD‐F patient and three PD‐NF patients with head motions exceeding 2.5 mm of translation or 2.5° of rotation were abandoned. Additionally, we calculated each subject's average framewise displacement (FD) and used it as a covariate in the subsequent comparison between DC groups. Intergroup comparisons did not reveal significant differences among the groups (*p* = 0.063). Then, after removing the scalp structure, 3D T1‐weighted images were registered to the functional images and subdivided into white matter (WM), gray matter (GM), and cerebrospinal fluid (CSF) by new segment and DARTEL technique, followed by segmenting into the nonlinear deformation space of the Montreal Institute of Neurology (MNI). We resampled the functional images with a resolution of 3 × 3 × 3 mm^3^, and used the Gaussian kernel (full width at half‐maximum, FWHM = 6 × 6 × 6 mm^3^) for spatially smoothing. Finally, bandpass filtering (0.01–0.08 Hz) was carried out, and the linear trend was removed. In fact, we also removed several noise covariates, including white matter noise signal, cerebrospinal fluid signal, and six head motion parameters obtained through head motion correction.

### 2.5 Voxel‐based DC analysis

Degree centrality data analysis is a voxel‐level assessment based on DPARSF. Pearson's correlation coefficient matrix was generated by calculating the temporal correlation between a gray matter mask voxel and all other voxels within the brain. Then, to eliminate the low time correlation interference caused by signal noise, we set the threshold of Pearson's correlation coefficient to *r* > 0.25.^10^, ^19^ Our study utilized binary DC values within the brain network to conduct subsequent statistical analyses. Afterwards, the fisher‐z transformation was served to convert the voxel‐wise DC values into a *z*‐score graph to enhance normality. Ultimately, all individual DC graphs were spatially smoothed together in the preprocessing stage (FWHM = 6 × 6 × 6 mm^3^).

### 2.6 Seed‐based FC analysis

Seed‐based FC analysis was conducted based on the brain regions with significant DC changes (compared PD‐F with PD‐NF group) and correlated with the mean FSS. We selected and defined the clusters (brain regions) as regions of interest (ROIs) for seeds. Before FC analysis, we first executed spatial smoothing (FWHM = 6 × 6 × 6 mm^3^). Next, for obtaining FC maps, we extracted ROIs time courses by averaging the time series of all voxels within the seed region and calculated the correlation between the ROIs and each voxel's average time series within the brain, including all voxels in the ROI itself. At last, we used Fisher's z transformation to convert FC maps into *z*‐score graphs to upgrade normality effectively.

### 2.7 Surface‐based Morphometry analysis

Surface‐based morphometry (SBM), carried out with CAT12, an SPM12 extension with the default pipeline (http://dbm.neuro.unijena.de/cat), was used for the cortical thickness analysis. Here, the distance measurement based on projection is employed to calculate the central surface, cortical thickness, and other indicators in barely one step.^20^ 3D T1‐weighted images were segmented to WM, GM, and CSF automatically; spherical registration to an MNI template space was applied; extracted and produced four SBM data, including cortical complexity, cortical thickness, and so on; resampled and smoothed surface as mentioned earlier by a 15 mm FWHM Gaussian kernel.

### 2.8 Statistical analysis

Data analysis was carried out based on IBM SPSS statistics 25.0 (SPSS). The Shapiro–Wilk test was performed to determine the data's normality. For normally distributed variables, one‐way analysis of variance (ANOVA) and two sample *t*‐test were adopted. For data that exhibited non‐normally distribution, we used non‐parametric test, including the Chi‐square test, Mann Whitney *U*‐test, and Kruskal Wallis test to compare sociodemographic and clinical data. Bonferroni correction was also performed. Statistical significance was defined as *p* < 0.05.

The statistical analysis module of DPABI software was operated to calculate and analyze the DC values of the three groups. We used the one‐way analysis of variance (ANOVA) to detect significant DC differences among the three groups, along with the covariates of sex, age, education, and mean FD. Based on the results of ANOVA, we subsequently performed a two‐sample post hoc *t*‐test with covariates described above between each pair among the three groups. All statistical significance complied with voxel‐wise *p* < 0.001 and a cluster‐level *p* value <0.05 after AlphaSim correction between groups.

Additionally, we extracted brain areas with varying DC values between PD subgroups as ROIs to perform FC analysis in the whole brain. ANOVA and post hoc *t*‐test were exerted to estimate FC differences between groups with sex, age, and education as covariates, combined with a voxel‐wise uncorrected *p* < 0.01 and an AlphaSim‐corrected cluster‐wise threshold of *p* < 0.01.

In order to clarify the association between brain regions with different DC and FSS scores, we commanded partial correlation analysis to calculate the correlation between the average DC value of the selected ROIs and the mean FSS score of PD‐F patients, taking the course of the disease, age of onset, LEDD, MMSE, HAMA, and HAMD‐24 scores as covariates to eliminate the interference of confounding factors. Two‐tailed *p* < 0.05 was considered a significant statistical difference. Subsequently, we utilized ROC curves to judge whether the extracted brain indicators could be regarded as PD‐F identification features. The cut‐off values' sensitivity, specificity, and area under the curve (AUC) were all reported. The optimal cut‐off value was selected by maximizing the Jordan index. Moreover, we also examined the Pearson's correlation between the average FC value and FSS score by SPSS 25.0 software to determine the correlation between the regions showing functional connectivity differences of ROIs and the severity of fatigue.

For SBM analysis, neural structure data was conducted on SPM12 and the CAT12 extension for inter‐group analysis. A single factorial analysis model was used, with sex, age, and education as covariates. Then, *t* contrasts were performed for inter‐group comparison based on ANOVA analysis. The significant level selected was *p* < 0.05.

## 3 RESULTS

### 3.1 Demographic and clinical characteristics

Clinical characteristics of the PD‐F group, PD‐NF group, and HCs group are listed in Table 1. The three groups observed no significant difference in age, sex, education, MMSE scores, and mean FD. We traced no statistical difference in the disease duration, age of onset, side of the beginning, modified H&Y stage, UPDRS‐III scores, LEDD, HAMA, HAMD‐24 scores, ESS, and AS scores between subgroups of PD (*p* > 0.05). However, PD‐F patients showed higher FSS scores than PD‐NF patients and HCs (*p* < 0.001). At the same time, members in the HCs group represented no fatigue.

**TABLE 1:** Demographic and clinical data of all subjects.

Items	PD‐F (*n* = 18)	PD‐NF (*n* = 20)	HCs (*n* = 23)	*p* Values	Post hoc (Bonferroni)
Age (years)	60.61 ± 8.20	65.05 ± 8.20	62.83 ± 4.96	0.170^a^	
Gender (F/M)	8/10	9/11	9/14	0.911^b^	
Education (years)	10.28 ± 4.03	10.20 ± 3.29	11.70 ± 3.04	0.281^a^	
MMSE	28.22 ± 1.15	28.40 ± 1.05	28.70 ± 0.97	0.560^c^	
Mean FD	0.10 ± 0.08	0.16 ± 0.10	0.13 ± 0.05	0.063^c^	
Disease duration (years)	6.50 ± 3.33	7.60 ± 3.39	NA	0.269^d^	
Age at onset (years)	52.33 ± 12.29	57.70 ± 8.52	NA	0.123^e^	
Affected side at onset right% (*n*)	55.6% (10)	55.0% (11)	NA	0.865^b^	
UPDRS‐III (OFF state)	32.560 ± 11.09	30.65 ± 12.70	NA	0.627^e^	
H&Y stages	2.42 ± 0.73	2.43 ± 0.52	NA	0.939^d^	
LEDD (mg/day)	686.27 ± 294.93	630.93 ± 218.04	NA	0.650^d^	
HAMA	10.39 ± 2.52	8.20 ± 3.65	NA	0.074^d^	
HAMD‐24	6.94 ± 3.39	5.45 ± 3.59	NA	0.212^d^	
ESS	6.61 ± 1.91	5.45 ± 2.82	NA	0.151^e^	
AS	10.89 ± 1.71	9.80 ± 2.12	NA	0.092^e^	
FSS/9	5.64 ± 1.16	2.46 ± 1.04	1.91 ± 0.67	<0.001^a^	*p* < 0.001^f^ ^,^ * *p* < 0.001^g^ ^,^ * *p =* 0.205^h^

*Note*: Values are presents as the mean ± standard deviation.

Abbreviations: AS, Apathy Scale; ESS, Epworth Sleepiness Scale; F, female; FSS, Fatigue Severity Scale; H&Y Stage Hoehn and Yahr Clinical Rating Scale; HAMA, Hamilton Anxiety Scale; HAMD‐24, Hamilton Depression Scale‐24; HCs, healthy controls; LEDD, levodopa equivalent daily dose; M, male; Mean FD, mean framewise displacement; MMSE, mini mental state examination; NA, not applicable; PD, Parkinson's disease; PD‐F, Parkinson's disease with fatigue; PD‐NF, Parkinson's disease without fatigue; UPDRS, Unified Parkinson's disease rating scale.

^a^ One‐way analysis of variance (ANOVA).

^b^ Chi‐square test.

^c^ Kruskal–Wallis test.

^d^ Mann–Whitney *U* test.

^e^ Two‐sample *t*‐test.

^f^ *p* Values between PD‐F and PD‐NF groups.

^g^ *p* Values between PD‐F and HCs groups.

^h^ *p* Values between PD‐NF and HCs groups.

* <0.05 was considered significant.

### 3.2 DC data and correlation analysis

**FIGURE 1:**
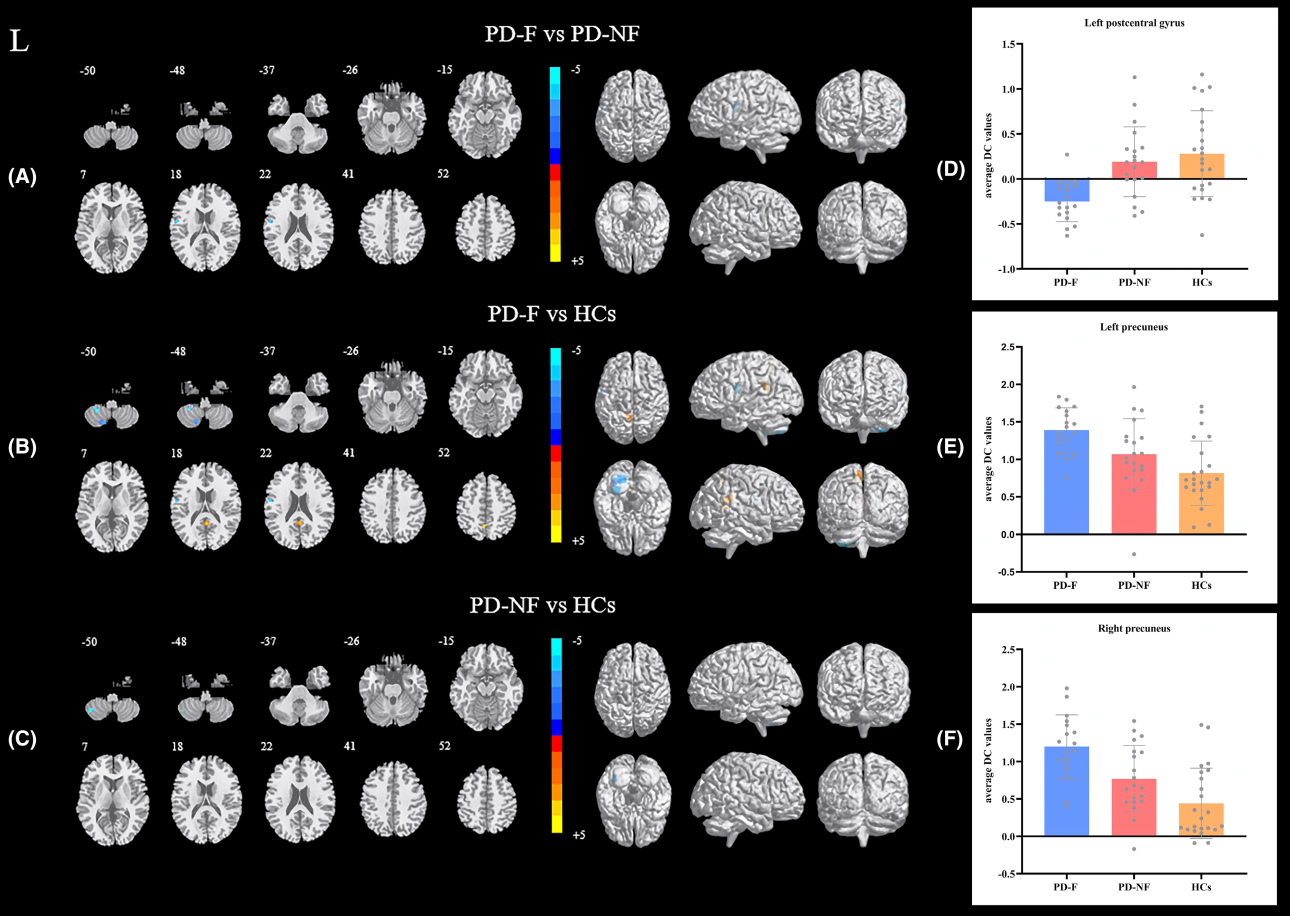
Significant differences of DC result maps and the distribution of DC values among three groups: PD‐F, PD‐NF, and HCs. Statistical threshold was displayed at voxel‐level *p* value <0.001, Alphasim corrected, cluster‐level *p* value <0.05. Warm colors represent significantly increased DC value and cool colors represent significantly decreased DC. (A) differences between PD‐F and PD‐NF; (B) differences between PD‐F and HCs; (C) differences between PD‐NF and HCs; (D) bar graphs of DC values distribution at the left postcentral gyrus among three groups; (E) bar graphs of DC values distribution at the left precuneus among three groups; (F) bar graphs of DC values distribution at the right precuneus among three groups. HCs, healthy controls; L, left; PD, Parkinson's disease; PD‐F, Parkinson's disease with fatigue; PD‐NF, Parkinson's disease without fatigue.

**FIGURE 2:**
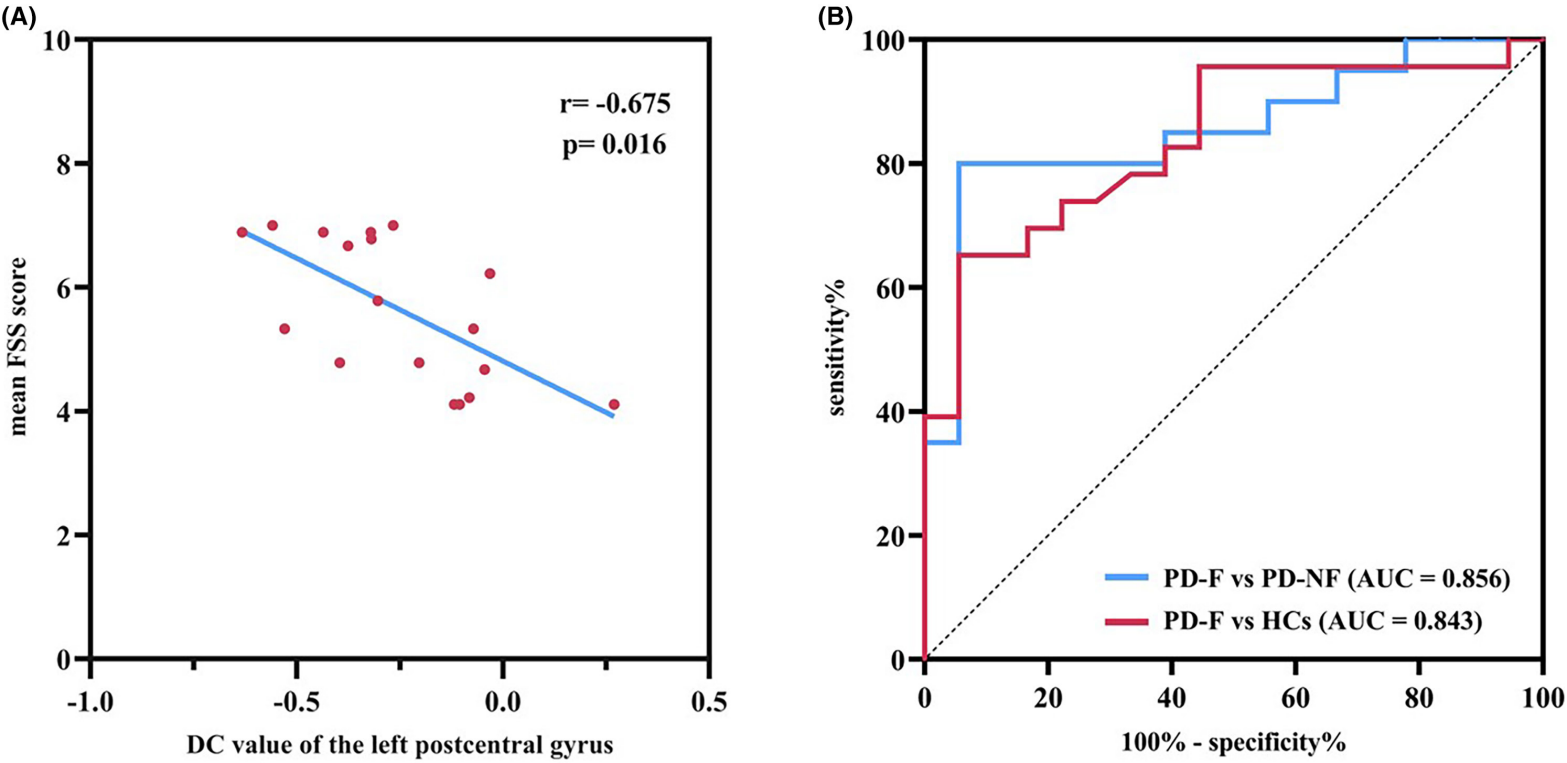
Correlations between DC value and mean FSS score in left postcentral gyrus during the OFF state of PD‐F patients and ROC analyses for differentiating different groups. (A) Scatterplots presented that there was a significant negative correlation between the DC value of the left postcentral gyrus and mean FSS score in PD patients with fatigue (*p* < 0.05). (B) The graph showed the results of the ROC analyses for differentiating different groups. The results revealed that the DC value of left postcentral gyrus shows significant potential as an indicator for separating PD‐F from PD‐NF (AUC = 0.856, *p* < 0.001) and an indicator for separating PD‐F from the HCs (AUC = 0.843, *p* < 0.001). AUC, area under the curve; DC, degree centrality; FSS, fatigue severity scale; HCs, healthy controls; PD, Parkinson''s disease; PD‐F, Parkinson''s disease with fatigue; PD‐NF, Parkinson''s disease without fatigue; ROC, receiver operating characteristic. See Table S1 for more details.

As exhibited in Figure 1 and Table 2, DC analysis presented that the DC value of the left postcentral gyrus decreased in PD‐F patients compared with PD‐NF patients. Further, increased DC values were observed in the bilateral precuneus lobes, whereas decreased DC values in the left postcentral gyrus and cerebellum lobule VIII region of the PD‐F patients compared with the HCs. Moreover, DC in the left cerebellum lobule VIIb was significantly reduced in the PD‐NF group versus the HCs. Our correlation analysis revealed that the average FSS scores and DC value of the left postcentral gyrus were negatively correlated (*r* = −0.675, *p* = 0.016; Figure 2A).

**TABLE 2:** Brain regions with abnormal DC between the groups.

Brain regions (AAL)	Peak MNI Coordinates			Number of voxels	Peak *T* value
	*x*	*y*	*z*		
PD‐F vs. PD‐NF					
Postcentral_L	−54	−3	18	13	−5.0841
PD‐F vs. HCs					
Postcentral_L	−54	−6	21	18	−5.5158
Precuneus_L	0	−54	51	27	5.2785
Precuneus_R	3	−48	18	39	4.9794
Cerebelum_8_L	−30	−45	−51	20	−5.5499
Cerebelum_8_L	−18	−69	−48	34	−4.2568
PD‐NF vs. HCs					
Cerebelum_7b_L	−45	−60	−51	14	−3.9920

*Note*: The threshold of post hoc *t*‐tests was set at voxel‐level *p* < 0.001 (before AlphaSim corrected), cluster‐level *p* < 0.05 (after AlphaSim corrected), determined by Monte Carlo simulation for multiple comparisons. Negative *T* value signifies the regions in which the former group had lower DC values than the latter group, while positive *T* value indicates the opposite.

Abbreviations: AAL, anatomical automatic labeling; DC, degree centrality; HCs, healthy controls; L, left; MNI, Montreal Neurological Institute; PD‐F, Parkinson's disease with fatigue; PD‐NF, Parkinson's disease without fatigue; R, right.

Furthermore, receiver operating characteristic (ROC) analysis showed that when distinguishing PD‐F patients from PD‐NF patients, the AUC of the left postcentral gyrus was 0.856 (95% confidence interval [CI]: 0.730–0.981, *p* < 0.001). Meanwhile, when distinguishing PD‐F patients from HCs, the AUC of the left postcentral gyrus was 0.843 (95% CI: 0.715–0.971, *p* < 0.001) (Figure 2B and Table S1).

### 3.3 FC data and correlation analysis

**FIGURE 3:**
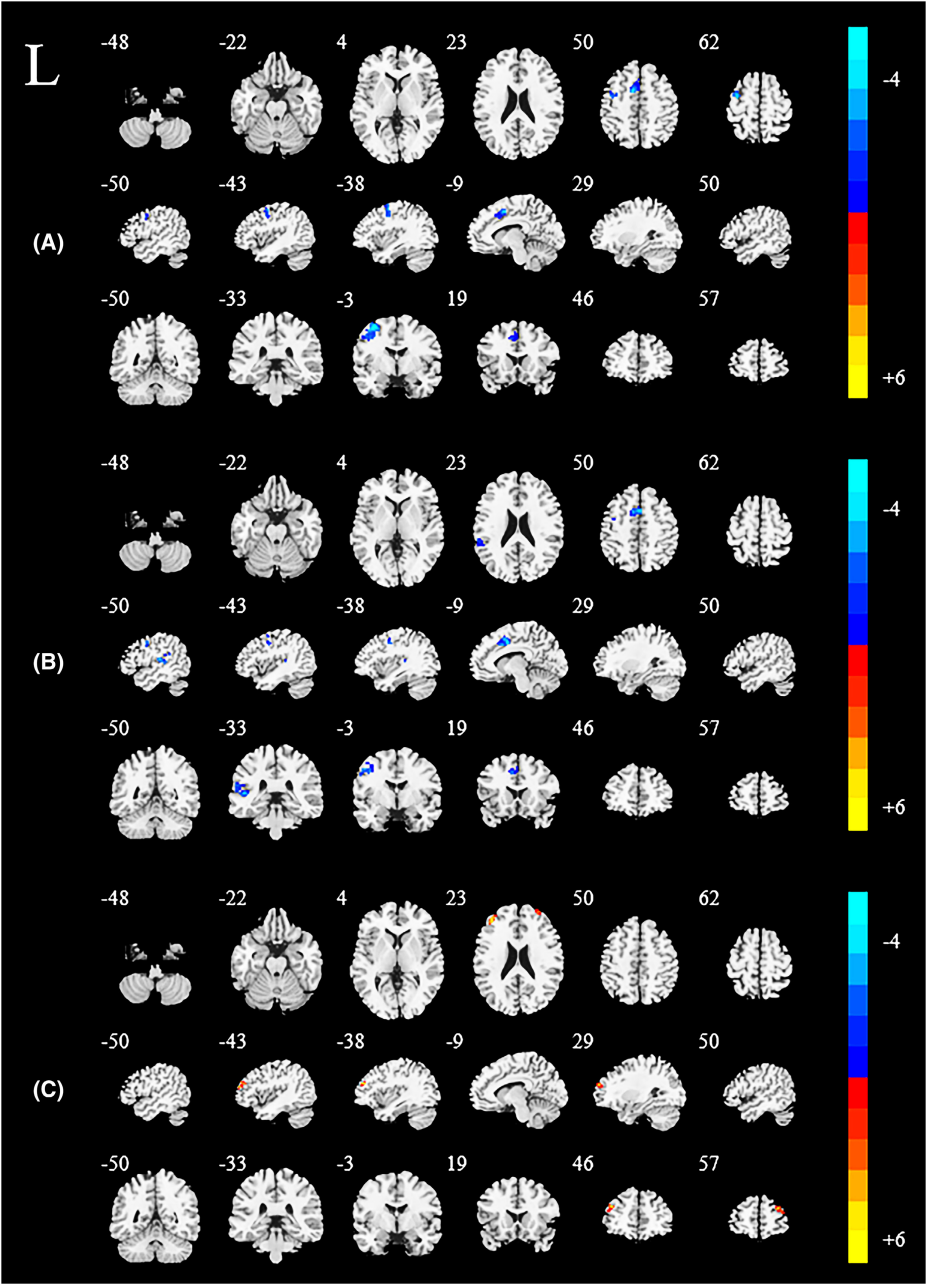
Significant differences in the FC of ROIs (left postcentral gyrus) among three groups: PD‐F, PD‐NF, and HCs. Statistical threshold was displayed at voxel‐level *p* value <0.01, Alphasim corrected *p* value <0.01. Warm colors represent significantly increased FC value and cool colors represent significantly decreased FC. (A) the left postcentral gyrus related FC between PD‐F and PD‐NF; (B) the left postcentral gyrus related FC between PD‐F and HCs; (C) the left postcentral gyrus related FC between PD‐NF and HCs. HCs, healthy controls; L, left; PD, Parkinson's disease; PD‐F, Parkinson's disease with fatigue; PD‐NF, Parkinson's disease without fatigue.

Regions showing significant differences in DC between the two subgroups of PD and associated with mean FSS scores were selected as the ROIs to detect the difference in brain network for FC analysis. The result was as follows: comparatively, the left postcentral gyrus displayed reduced FC values with the left supplementary motor area (SMA) and the left precentral gyrus in the PD‐F group versus the PD‐NF group. While comparing with HCs, in the PD‐F group, the left postcentral gyrus exhibited lower FC in the left middle cingulate cortex (MCC), left precentral gyrus, and left superior temporal gyrus. No increased FC values were observed in the PD‐F group. Nevertheless, in the PD‐NF group, the left postcentral gyrus had increased FC values with the bilateral middle frontal gyrus (MFG) than the HCs group (Figure 3 and Table 3).

**TABLE 3:** Brain regions with abnormal FC based on the ROIs (left postcentral gyrus) between the groups.

Brain regions (AAL)	Peak MNI Coordinates			Number of voxels	Peak *T* value
	*x*	*y*	*z*		
PD‐F vs. PD‐NF					
Supp_Motor_Area_L	−9	6	51	112	−5.8059
Precentral_L	−33	−3	60	120	−6.6404
PD‐F vs. HCs					
Cingulum_Mid_L (extending to Supp_Motor_Area_L)	−9	3	42	143	−4.7269
Precentral_L	−48	−3	45	42	−4.0497
Temporal_Sup_L	−57	−42	18	97	−5.0056
PD‐NF vs. HCs					
Frontal_Mid_L	−39	45	24	26	4.7117
Frontal_Mid_R	30	57	21	30	4.0534

*Note*: The threshold of post hoc *t*‐tests was set at voxel‐level *p* < 0.01 (before AlphaSim corrected), cluster‐level *p* < 0.01 (after AlphaSim corrected), determined by Monte Carlo simulation for multiple comparisons. Negative T value signifies the regions in which the former group had lower FC values than the latter group, while positive T value indicates the opposite.

Abbreviations: AAL, anatomical automatic labeling; FC, functional connectivity; HCs, healthy controls; L, left; MNI, Montreal Neurological Institute; PD‐F, Parkinson's disease with fatigue; PD‐NF, Parkinson's disease without fatigue; R, right.

It is worth mentioning that the correlation analysis between the left postcentral gyrus and the left SMA‐related FC network alterations negatively correlated with the mean FSS score of PD‐F patients when compared with the PD‐NF group (*r* = −0.526, *p* = 0.025). The FC values between the left postcentral gyrus and precentral gyrus had similar correlation outcomes (*r* = −0.478, *p* = 0.045; Figure S1).

### 3.4 SBM analysis

No statistical difference among the three groups for the cortical thickness at a threshold of FWE‐corrected *p* < 0.05 was detected in this study.

## 4 DISCUSSION

Our study indicated that the altered functional connectivity in the sensorimotor network centering on the left postcentral gyrus and the bilateral precuneus might be a potential pathogenic mechanism of PD patients with fatigue. More specifically, as opposed to the PD‐NF and HCs groups, the PD‐F group presented lower DC in the left postcentral gyrus. Next, the FSS exhibited a negative correlation with the DC value in the left postcentral gyrus. Additionally, the ROC curves supported that the DC value of the left postcentral gyrus might be a promising neuroimaging indicator for identifying individuals with PD‐F. Our FC analysis further revealed that brain network interruption regions with the left postcentral gyrus in PD‐F patients mainly involved the left precentral gyrus and the SMA region. Second, the PD‐F group showed higher DC in the bilateral precuneus than the HCs group. Third, both PD subgroups demonstrated reduced DC values in the specific region of the cerebellum. Finally, we discovered no statistical difference in cortical thickness among the three groups, which implied that observed FC abnormalities were independent from structural changes in cortical thickness. Overall, our research provided theoretical guidance for a better understanding of fatigue in PD.

Research shows that aberrant sensory attenuation is an independent mediator of fatigue. When abnormal sensory attenuation occurs, the perception of effort increases accordingly, affecting the spontaneous neuronal firing in the resting state and ultimately making the body tired.^21^ A structural MRI study of fatigue in ankylosing spondylitis supported the above viewpoint, finding that more significant fatigue was associated with thinning in the somatosensory cortex.^22^ Such observations were in good accordance with our findings that PD patients with fatigue exhibited decreased DC value in the left postcentral gyrus, suggesting a reduced node density and information transmission function at this location in the brain network. Moreover, we further detected that the abnormally functional connection with this seed point mainly involved the left SMA and precentral gyrus. SMA integrated and encoded the information received from the sensory cortex and directly transmitted to the primary motor cortex (PMC) of the precentral gyrus.^23^ On this basis, we reasonably hypothesize that sensory input attenuation leads to altered connections between the sensory cortex and the higher‐order motor cortex, such as the SMA, PMC, eventually resulting in fatigue in PD patients. FC analysis of PD fatigue in drug‐naïve patients also found that fatigue was relevant to diminished connectivity of SMA in sensorimotor networks.^8^ Correspondingly, a global FC analysis across brain networks revealed that lower connectivity in the central sensorimotor system seems to be linked explicitly to fatigue in multiple sclerosis patients.^24^ Moreover, transcranial direct current stimulation (tDCS) and other related stimulation on the bilateral somatosensory cortex could effectively combat fatigue in patients with multiple sclerosis.^25^, ^26^, ^27^ In line with previous reports, our correlation analysis exposed that fatigue severity in PD was related to the destruction of functional connections in the sensorimotor network. Furthermore, ROC analysis implied that DC value in the postcentral gyrus could discriminate PD‐F patients from PD‐NF patients and HCs. Altogether, our investigation supported the view that the decreased DC in the left postcentral gyrus we discovered demonstrated impaired information transmission function in the PD‐F group, which destroyed the FC of the sensorimotor network centering on the postcentral gyrus, causing the fatigue symptom in PD patients. However, several researchers reported opposite results, specifically increased connectivity between the left pre‐SMA and the left postcentral gyrus in drug‐naïve PD patients with fatigue.^9^ We speculated that this might be due to a compensatory response in the early stage of the disease, whereas compensation gradually transforms into the decompensation stage as the pathology progress,^28^ which needs to be further verified by longitudinal research studies in PD fatigue.

The precuneus, an essential node of the DMN,^29^ has been proven to be involved in visual perception, episodic memory retrieval, self‐processing, and consciousness.^30^ Our findings identified increased DC values of the bilateral precuneus in PD‐F patients in comparison with HCs, which was deduced to compensate for the decreased sensorimotor network connectivity in PD‐F patients by enhancing their functional connectivity. In fact, the precuneus, which serves as a compensatory region by enhancing signal synchronization, has been observed in cognitive impairment and freezing of gait in PD.^12^, ^31^, ^32^ A previous fMRI study also reported that fatigued multiple sclerosis patients demonstrated decreased precentral and postcentral gyrus activation and increased precuneus activation, confirming that fatigued multiple sclerosis patients were likely to maintain overrecruit of the precuneus to adapt to the increased task demands.^33^ A further explanation might be that, as a core component of the DMN, the activation of the precuneus represented compensatory effects of the DMN, which is responsible for the cognitive function of self‐referential introspection.^34^ Of course, this was consistent with the cognitive compensation proposed by the previous FC study on fatigue in drug‐naïve PD patients.^8^ However, we could not rule out the change in the precuneus function as a driving factor that leads to body fatigue. In other words, the higher precuneus connectivity of the DMN in PD‐F patients resulted in the excessive generation of inner thoughts, inevitably bringing more energy consumption of the brain,^35^, ^36^ thus exacerbating PD fatigue. These results strengthen the viewpoint that the precuneus is a crucial hub involved in the mechanism of fatigue in PD. Interestingly, we did not observe the difference in DC values of the precuneus between the PD‐F and the PD‐NF groups. Therefore, we had reason to believe that the alternation was not significant between PD subgroups by increasing mental effort expenditure to sustain the same level of motor performance.

Interestingly, the majority of our results were primarily located in the left hemisphere, which may imply lateralization of pathological localization in PD patients with fatigue. The possible reason for this was that our subjects were all right‐handed, and the left hemisphere was the dominant hemisphere. Similarly, a magnetoencephalography study found that physical fatigue was associated with the left sensorimotor area,^37^ which supported our conjecture. Furthermore, our previous study also demonstrated that interhemispheric functional incoordination might participate in the pathogenic mechanism of fatigue in PD, suggesting fatigue correlated with brain lateralization.^38^ Unfortunately, we did not find differences in the structure of fatigued PD patients. Moreover, there is no clear and consistent evidence of functional or pathologic localization of lateralization in PD patients with fatigue.

Our research had some limitations. First of all, the sample size of patients included in this study was relatively small, affecting its representativeness. Nevertheless, our results were still trustworthy because they survived multiple comparisons correction, and we eliminated the interference of anxiety, depression, sleep, and so forth. Second, we used the clinical scale to evaluate the degree of fatigue in this study, lacking an objective measurement standard despite the availability and reliability of FSS that has been proved.^39^, ^40^ Third, this study was a cross‐sectional study. At present, it might not be clear whether the findings of this study were adaptive changes or maladjustment. The current evidence was insufficient to clarify the causal relationship between changes in brain regions and fatigue, which should need further longitudinal research. Therefore, we may consider expanding the sample size for a future longitudinal follow‐up study.

## 5 CONCLUSION

To conclude, our results showed that PD‐F patients represented abnormal network connection density in the postcentral gyrus and aberrant FC in the sensorimotor network centering on the left postcentral gyrus and the bilateral precuneus. The findings also uncovered that these alternations were predictive factors of fatigue severity. In short, anomalous functional connectivity in the sensorimotor network system and the bilateral precuneus are closely related to fatigue in PD patients. The left postcentral gyrus is expected to be the potential imaging marker of PD fatigue.

## AUTHOR CONTRIBUTIONS

AS, HZ, MG, LW, XC, CG, and HS jointly completed the research project and the statistical analysis. AS wrote the first draft of the article. MG checked the article. HZ checked and revised the first draft. KZ and YY designed this study and revised the manuscript for intellectual content. All authors approved the final manuscript.

## FUNDING INFORMATION

This work was supported by the National Natural Science Foundation of China (grant numbers 81671258 and 81901297).

## CONFLICT OF INTEREST STATEMENT

The authors declare that there is no conflict of interest.

## Supporting information


Figure S1
Table S1

## Data Availability

The original contributions presented in the study are included in the article/supplementary material; further inquiries can be directed to the corresponding authors.
