# Supplementary material for: Aberrant voxel‐based degree centrality and functional connectivity in Parkinson's disease patients with fatigue
Source: CNS Neurosci Ther. 2023 Apr 10;29(9):2680–9. doi: 10.1111/cns.14212 (PMC10401083; doi:10.1111/cns.14212)
Supplement: Supplementary file 1 — Figure S1 Table S1 [file CNS-29-2680-s001.docx]

**Supplementary material**

**Table 1** ROC analyses for differentiating different groups.

| **Brain regions** | **AUC** | ***p* value** | **95% CI** | **Sensitivity** | **Specificity** | **Cut-off point** |
| --- | --- | --- | --- | --- | --- | --- |
| **Postcentral_ L** | | | | | | |
| Separating PD-F from PD-NF | 0.856 | < 0.001^**^ | 0.730-0.981 | 0.800 | 0.944 | -0.0190 |
| Separating PD-F from HCs | 0.843 | < 0.001^**^ | 0.715-0.971 | 0.700 | 0.944 | 0.0003 |
| Separating PD-NF from HCs | 0.506 | 0.953 | 0.317-0.694 | 0.300 | 0.833 | 0.3785 |

*ROC* receiver operating characteristic, *AUC* area under the curve, *CI* confidence interval, *PD-F* Parkinson’s disease with fatigue, *PD-NF* Parkinson’s disease without fatigue, *HCs* healthy controls.

*** p* < 0.001.

**
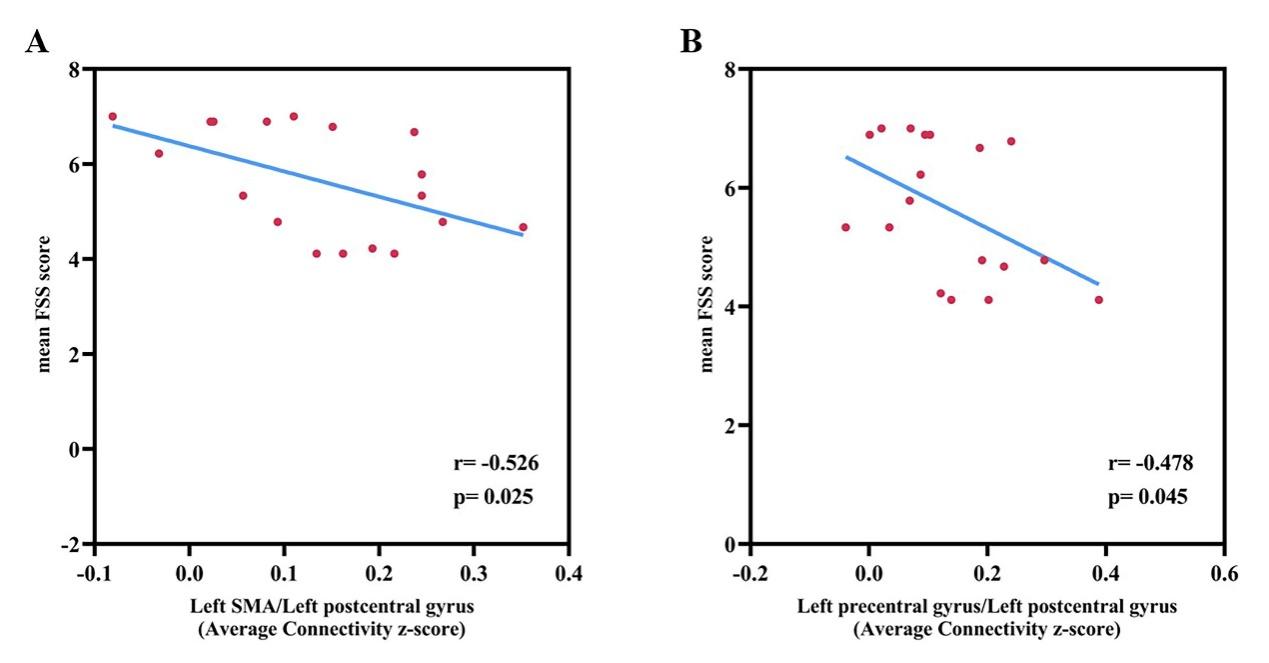
**

**Figure 1** Correlations between FC value and mean FSS score based on ROIs (left postcentral gyrus) during the OFF state of PD patients with fatigue. (A) Correlation analysis between the mean fatigue severity scale score and functional connectivity measure (Average Connectivity z-score) between left SMA and left postcentral gyrus in PD-F patients (*p* < 0.05). (B) Correlation analysis between the mean fatigue severity scale score and functional connectivity measure (Average Connectivity z-score) between left precentral gyrus and left postcentral gyrus in PD-F patients (*p* < 0.05).
